# Radiotherapy of Oligometastatic Cancers

**DOI:** 10.3390/cancers16030511

**Published:** 2024-01-24

**Authors:** Filippo Alongi, Simona Gaito

**Affiliations:** 1 Advanced Radiation Oncology Department, IRCCS Sacro Cuore Don Calabria Hospital, Cancer Care Center, Negrar, 37024 Verona, Italy; 2 University of Brescia, 25121 Brescia, Italy; 3 Department of Clinical Oncology, The Royal Marden Hospital, London SW3 6JJ, UK; 4 Division of Medical Science, The University of Manchester, Manchester M13 9PL, UK

The enhanced multidisciplinary treatment approach and the widespread use of advanced imaging techniques have led to an improvement in survival rates, inevitably associated with an increase in the number of oligometastatic diagnoses in cancer patients. In this clinical setting, systemic therapies such as chemotherapy, targeted therapy, immuno-therapy, and hormone therapy are considered the most effective and widely accepted treatment options. Metastasis-directed therapies (MDTs) have been studied in both retrospective analyses and prospective trials as a potential treatment option to improve progression-free survival or delay the use of systemic treatments in various scenarios, such as oligoprogressive/oligopersistent cancer disease. This is because MDTs have shown promising results in cases with limited metastatic burden and often lead to favourable outcomes.

In the context of oligometastatic disease, as proposed by Hellman and Weichselbaum [1], radiation therapy (RT) is a compelling treatment option for the control of oligometastases. At an MDT level, there is general consensus on using RT as a way of prolonging the disease-free interval and improving quality of life. Stereotactic body radiation therapy (SBRT), which is also known as stereotactic ablative radiation therapy (SABR), is the gold standard when it comes to MDRT (metastasis-directed RT). This treatment can be administered with or without systemic therapy. Moreover, there is emerging clinical evidence that supports the use of highly advanced and targeted RT in this clinical scenario. The latest findings from the SABR-COMET trial [2], which have been recently updated, validate the benefits of adding SBRT to the standard-of-care treatment (as dictated by the tumour histology and stage), resulting in improved survival rates. These results align with the consistent data from other trials that demonstrate the promising use of SBRT in treating oligometastases. However, there are still several objections and unresolved issues that continue to be subjects of ongoing debate. Although MDRT has been widely implemented in the clinical practice of treating oligometastases, there is still significant diversity in definitions of the optimum candidate, the appropriate RT doses and schedules, and the volumes to be irradiated, as well as in selecting treatment planning optimisation methods. Moreover, published patient reported outcomes (PROMs) are lacking in patients treated for oligometastatic disease, and there is a need for more granular collection of this data through health-related quality of life questionnaires to better understand the magnitude of the effect of SBRT/SBRT [3].

In this Special Issue dedicated to RT in oligometastatic cancer, Vorbach and colleagues [4] evaluated the safety and effectiveness of SBRT in the largest published cohort of exclusively oligometastatic lung diseases derived from head and neck squamous cell carcinoma (HNSCC). SBRT was reported as a safe and effective treatment alternative to surgical resection for inoperable patients and/or unresectable lesions. In the 92 lung oligometastases, excellent local control was reported, even after repeated SBRT courses of newly progressive metastases. The overall survival of the patient population was in the upper range of previously published results. Toxicities was limited, and no functional lung impairment was reported, confirming the emerging role of RT in lung oligometastatic well-selected head and neck patients.

Marvaso et al. [5], of the AIRO oligometastatic study group, reported how RT has contributed to shifting the paradigm in oncology over the last two decades; they did so, using a bibliometric analysis of the oligometastatic scenario. Their study represents a detailed summary of the most influential studies addressing oligometastatic status. More specifically, a total of 3304 published manuscripts were collected from 1995 to 2022. Among the several keywords employed by authors, the three most frequently utilised were oligometastases (19%), SBRT (18%), and radiation therapy (8%). Furthermore, a crosslink was shown between RT and subjects such as immunotherapy and targeted therapies; such a crosslink presents new avenues for investigation into therapeutic alternatives and combination strategies in this context.

Pastorello et al. [6] reported their experience using SBRT in the treatment of 150 non-spinal bone oligometastases in 95 prostate cancer cases. The treatment was delivered to patients with macroscopic disease defined using ^68^Ga-PSMA-PET/CT and CT. Considering the limited number of local relapses (eight cases), their results provide further evidence supporting the use of ablative focal radiotherapy in non-spinal bone oligometastases from prostate cancer.

The research group of Zapatero and colleagues [7] explored the challenging issue of nodal oligorecurrence in prostate cancer. Their manuscript aims to summarize the data supporting this approach, discussing controversies concerning patient selection and the best treatment option, reporting both ongoing phase III trials and the future of nodal recurrence in approaches to prostate cancer. In fact, because of its indolent behaviour as well as the significant breakthroughs in molecular imaging such as PSMA-PET, the investigation of oligonodal failure remains a subject of intensive research. As a result, MDRT using SBRT is also becoming an increasingly attractive treatment opportunity for these patients.

Gaito et al. [8] investigated the potential role of proton therapy (PT) technology for the treatment of oligometastatic disease with hypofractionation. While conventional photon RT is a well-established treatment option for oligorecurrent/oligometastatic patients, the role of PT in this setting remains to be defined. The available evidence was reviewed, with particular attention given to the potential advantages and disadvantages of PT in specific clinical scenarios and to the opportunity offered by the latest pencil-beam scanning technology to overcome some of the current dosimetric concerns around the use of PT in this setting.

In summary, the goal of the present Special Issue is to discuss the emerging role of RT in oligometastatic cancer, that we might advance our understanding of the disease biology and maximise the benefits of RT in this increasingly more frequent clinical scenario.
